# Editorial note: flying high in Japan

**DOI:** 10.1080/19336934.2023.2173997

**Published:** 2023-03-06

**Authors:** Ryusuke Niwa, Makoto Sato, Tatsushi Igaki

**Affiliations:** Life Science Center for Survival Dynamics, Tsukuba Advanced Research Alliance (TARA), University of Tsukuba, Ibaraki, Japan; Institute for Frontier Science Initiative, Kanazawa University, Ishikawa, Japan; Laboratory of Genetics, Graduate School of Biostudies, Kyoto University, Kyoto, Japan

In 1922, Professor Yoshimaro Tanaka at Kyushu University introduced *Drosophila melanogaster* as a model organism for research in Japan, importing the flies from the University of California [1]. Today, 100 years after this initial step, it is estimated that approximately five or six hundred Japanese people work in *Drosophila* research, based on the registered information on the Japanese *Drosophila* researcher mailing list, JFly (https://jfly.shigen.info/?lang=en).

As a community platform primarily for Japanese *Drosophila* researchers, the Japanese Drosophila Research Conference (JDRC) has been held almost biennially since 1993. In recent years, the conference typically attracts around 300 attendees. While the conference may not be as large as the Annual Drosophila Research Conferences organized by the Genetics Society of America, attendees can experience a diverse range of presentations, covering disciplines from molecular biology to ecology and research topics from embryos to adults, from development to diseases, featuring not only *D. melanogaster* but also other *Drosophila* species.

Currently, the JDRC is organized by ten organizing committee members (listed in https://gakujutsushukai.jp/jdrc15?locale=en). Among the committee members, three (R.N., M.S., and T.I.) are also Editorial Board members of the journal *Fly*. Since we three on the Editorial Board were commissioned by the journal to compile a special collection on current *Drosophila* research in Japan, we decided to organize it as a special section in volume 16 of the journal, featuring ten topics presented at the 14th JDRC held in September 2021, of which R.N. was the chief organizer. A summary of each review article is presented below. The following order is based on the alphabetical order of the contributing PIs’ family names.

T.I. (Tatsushi Igaki, Kyoto University) and his colleague summarize cell-cell interactions in the tumour microenvironment that play a crucial role in tumorigenesis [2]. *D. melanogaster* allows us to utilize genetic mosaic techniques that have provided a powerful platform to study the role of cell-cell interactions in tumorigenesis. Many studies using *D. melanogaster* have identified oncogenic cell-cell interactions triggered by various factors, including microenvironmental factors that influence tumour growth and metastasis. The authors review recent advances in such interactions at the cellular level, which may directly contribute to the understanding of tumour progression in humans.

Dr. Yuki Ishikawa and her colleagues introduce the interesting non-*melanogaster* species, *D. elegans* and its close relatives, which are useful model organisms for understanding mechanisms of adaptive evolution [3]. These species exhibit unique characteristics such as flower-breeding preferences, courtship display, territoriality, sexual dimorphism, and colour polymorphism. The ease with which they can be cultured and the availability of genomic information make them useful for evolutionary studies. Recent research has revealed their unique mating and territorial behaviours, characteristic flower-dependent biology, food habits, and life-history traits, as well as their distinctive karyotype and genetic mechanisms of morphological diversity. As far as we know, the paper is the first review to focus on *D. elegans* and its relatives.

Dr. Yutaka Matsubayashi (Bournemouth University, United Kingdom) reviews multiple lines of evidence that extracellular matrices (ECMs) can undergo rapid changes of organization and composition in *D. melanogaster* and other organisms [4]. The paper also highlights how cutting-edge technologies have contributed to revealing the previously invisible dynamics of ECMs. ECM dynamics is emerging as a hitherto unrecognized critical factor for tissue development and maintenance.

Dr. Yu-ichiro Nakajima (University of Tokyo) and his colleagues describe the mechanism underlying adult tissue plasticity in *D. melanogaster* [5]. Adult tissue plasticity allows for dynamic remodelling of structures in response to environmental challenges, preventing tissue dysfunction and improving organism fitness. Studies using *D. melanogaster* have provided insight into molecular mechanisms that control cellular responses in regeneration and nutrient adaptation, with a focus on stem-cell proliferation, polyploidization, and cell-fate plasticity in various adult organs. The review discusses the organismal strategy against environmental change and future research directions.

Dr. Naoki Okamoto (University of Tsukuba) and his colleague summarize recent advances in understanding the target organs/tissues and functions of peripherally derived peptide hormones in *D. melanogaster* [6]. They also describe how these hormones contribute to various biological events through interorgan communication. The authors also show how *D. melanogaster* serves as an excellent model system for understanding interorgan communications through endocrine factors, given the conservation of essential endocrine systems between flies and mammals and the availability of powerful genetic tools.

M.S. (Makoto Sato, Kanazawa University) and his colleague review recent technical advances for the study of the temporal regulation of neurogenesis in *D. melanogaster* [7]. The central nervous system (CNS) of *D. melanogaster* has been used for a long time as an important model in understanding the mechanisms that control neurogenesis spatiotemporally. Recent studies have shown that transcription factors are sequentially expressed in neural progenitor cells to regulate the temporal specification of neurons in the CNS. The paper also summarizes omics analysis using single-cell RNA-seq and other methods which have been used to study the *D. melanogaster* CNS on a large scale. These technologies make a significant contribution to understanding the temporal mechanisms of neurogenesis.

Dr. Atsushi Sugie (Niigata University) and colleague describe the use of *D. melanogaster* in neurodegenerative disease research, which has led to the identification of essential genes for human pathology [8]. The compact structure of the brain in *D. melanogaster* and the conservation of genes across species allow for rapid genetic analysis, revealing pathological mechanisms at the molecular level that are involved in human neurological diseases. Recent ‘reverse translational research’ using *D. melanogaster*, i.e. where findings from pathological studies in humans inform experimental studies in the fly, are discussed, and various methods for quantifying neurodegeneration in *D. melanogaster* are introduced in this review.

Dr. Daiki Umetsu (Tohoku University) focuses on the novel roles of Toll-related receptors (TRRs) in *D. melanogaster* [9], where they play a crucial role in tissue morphogenesis and homoeostasis. They are essential for innate immunity and have diverse functions in various biological processes. TRRs are also found to directly regulate cell mechanics and cell-cell recognition independently of the canonical signal-transduction pathway whereby they influence the transcriptional regulation of target genes. The manuscript summarizes recent advances revealing their non-immune functions in the control of epithelial tissue homoeostasis, tissue morphogenesis, and cell-cell recognition between different cell identities.

Dr. Tomoko Yamakawa (Osaka University) and her colleagues focus on the role of Notch signalling in the maternal-to-zygotic transition (MZT), a developmental phase during which maternal transcripts and proteins in the egg are cleared and zygotic genome transcription is activated [10]. Notch signalling is a highly conserved pathway across metazoans that regulates various developmental processes, and its defects are commonly associated with human diseases. In many organisms, including *D. melanogaster*, genes involved in Notch signalling are extensively influenced by the MZT. The authors have recently identified and characterized maternal genes involved in Notch signalling, namely *pecanex* and *almondex*. The review focuses on the roles of these two maternal genes in Notch signalling.

Lastly, Tetsuo Yasugi (Kanazawa University), along with M.S., also concentrates on the study of Notch-dependent neural development in *D. melanogaster* [11]. Typically, Notch signalling plays a crucial role in regulating lateral inhibition to specify embryonic neural stem cells and sensory-organ precursors in the thorax. Additionally, it has been found to collaborate with other signalling pathways to regulate the optimal positioning of photoreceptor cells in the eyes and the timing of neural stem-cell differentiation in the optic lobe. To understand the intricate interplay between Notch signalling and other pathways, mathematical analysis has been essential. The paper summarizes how theoretical and computational studies have been instrumental in revealing the dynamics of Notch signalling in these systems.

We believe that these ten reviews, although not spanning the entire diversity of *Drosophila* research in Japan, cover a relatively broad range of topics, from embryonic development to adult physiology and disease, and from molecules to ecology. We hope that this special collection will provide an opportunity for the global fly community to become more familiar with, and interested in, the work of *Drosophila* researchers in Japan. In addition, we warmly welcome all our colleagues from overseas to participate in future JDRC meetings. The next JDRC will be held in Sendai, Japan, organized by Dr. Erina Kuranaga (Tohoku University) in September 2024!
